# Differential Cytotoxic Activity of a Novel Palladium-Based Compound on Prostate Cell Lines, Primary Prostate Epithelial Cells and Prostate Stem Cells

**DOI:** 10.1371/journal.pone.0064278

**Published:** 2013-05-10

**Authors:** Engin Ulukaya, Fiona M. Frame, Buse Cevatemre, Davide Pellacani, Hannah Walker, Vincent M. Mann, Matthew S. Simms, Michael J. Stower, Veysel T. Yilmaz, Norman J. Maitland

**Affiliations:** 1 Department of Medical Biochemistry, Medical School, Uludag University, Bursa, Turkey; 2 Department of Biology, YCR Cancer Research Unit, University of York, Heslington, York, North Yorkshire, United Kingdom; 3 Department of Biology, Faculty of Arts and Sciences, Uludag University, Bursa, Turkey; 4 Hull York Medical School, University of Hull, Hull, United Kingdom; 5 Department of Urology, Castle Hill Hospital (Hull and East Yorkshire Hospitals NHS Trust), Cottingham, United Kingdom; 6 York District Hospital, York, United Kingdom; 7 Department of Chemistry, Faculty of Arts and Sciences, Uludag University, Bursa, Turkey; Univ of Bradford, United Kingdom

## Abstract

The outcome for patients with advanced metastatic and recurrent prostate cancer is still poor. Therefore, new chemotherapeutics are required, especially for killing cancer stem cells that are thought to be responsible for disease recurrence. In this study, we screened the effect of a novel palladium-based anticancer agent (Pd complex) against six different prostate cancer cell lines, and primary cultures from seven Gleason 6/7 prostate cancer, three Gleason 8/9 prostate cancer and four benign prostate hyperplasia patient samples, as well as cancer stem cells selected from primary cultures. MTT and ATP viability assays were used to assess cell growth and flow cytometry to assess cell cycle status. In addition, immunofluorescence was used to detect γH2AX nuclear foci, indicative of DNA damage, and Western blotting to assess the induction of apoptosis and autophagy. The Pd complex showed a powerful growth-inhibitory effect against both cell lines and primary cultures. More importantly, it successfully reduced the viability of cancer stem cells as first reported in this study. The Pd complex induced DNA damage and differentially induced evidence of cell death, as well as autophagy. In conclusion, this novel agent may be promising for use against the bulk of the tumour cell population as well as the prostate cancer stem cells, which are thought to be responsible for the resistance of metastatic prostate cancer to chemotherapy. This study also indicates that the combined use of the Pd complex with an autophagy modulator may be a more promising approach to treat prostate cancer. In addition, the differential effects observed between cell lines and primary cells emphasise the importance of the model used to test novel drugs including its genetic background, and indeed the necessity of using cells cultured from patient samples.

## Introduction

Prostate cancer is the most commonly diagnosed cancer in males and is the second highest cause of male cancer-related death [1], [2]. Although new drugs have recently been introduced into the clinic, the response to therapy for metastatic prostate cancer is still poor [3], [4], [5]. Therefore, there is an urgent need for more efficient or different kinds of drugs specifically targeting radio-recurrent and hormone-resistant prostate cancer, as well as prostate cancer stem cells (CSCs) [3], [4], [6]. New metal-based agents like palladium (Pd) complexes are promising for the development of improved chemotherapeutic drugs. There is a significant similarity between the coordination chemistry of Pd and platinum (Pt) compounds as antitumor drugs [7].

Although the synthesis of Pd complexes with anti-fungal, anti-viral, anti-cancer, and anti-bacterial activities dates back to more than 30 years [8], the anti-cancer activities of Pd complexes have become of increasing interest within the last 15 years. As such, different Pd complexes with promising activity against varying kinds of tumor cell lines from both solid tumors and hematological malignancies have been synthesized and tested over the years [9], [10], [11], [12], [13], [14], [15]. Their lipophilicity or solubility seems to provide satisfactory cytostatic activity [16]. The increased solubility of Pd complexes, compared to platinum, also makes Pd complexes more attractive. For example, Pd complexes of glyoxylic oxime were found to have higher aqueous solubility than platinum(II) (Pt) complexes of glyoxylic oxime [17].

There are only a few studies on the effect of newly-synthesized palladium(II) complexes on prostate-derived cell lines: for example, palladium(II) has been complexed with different ligands such as triazole [10], triphenylphosphines [18], dithiocarbamate [19], or hydrazine [20]; and even curcumin, which is a well-known plant-based compound with apoptosis-inducing activity on cancer cells [21].

In addition to the ligands above, the bioorganic and medicinal chemistry of 2,2′:6′,2″-terpyridine (terpy) complexes of Pd(II) and Pt(II) is also an active and growing area of interest [13], [22]. Taking into account the promising activity of Pd complexes against cancer, we have therefore synthesized new Pt and Pd complexes; [Pd(sac)(terpy)](sac)·4 H_2_O, [Pt(sac)(terpy)](sac)·5 H_2_O, [PdCl(terpy)](sac)·2 H_2_O, [PtCl(terpy)](sac)·2 H_2_O (sac = saccharinate, and terpy = 2,2′:6′2″-terpyridine) [23]. Among these, the Pd complexes, but not the Pt complexes, were found to exhibit considerable anti-growth effect against non-small cell lung cancer cells in vitro [24]. The [Pd(sac)(terpy)](sac)·4 H_2_O complex was further investigated against breast cancer cells both in vitro and in vivo and showed powerful anti-growth activity against this cancer type [25].

In the present paper, we have investigated the cytotoxic activity of our formulation of Pd complex, formulated as [PdCl(terpy)](sac)·4 H2O, against prostate cancer cells. The Pd complex was found to exhibit powerful growth-inhibiting activity, against cell lines and primary cultures, as well as prostate CSCs. The induction of apoptosis in cell lines by this compound indicates its potential as a new cytotoxic agent. However, the induction of autophagy but not apoptosis in primary prostate cells suggests that a combination of the complex with autophagy inhibitors may be a preferred treatment strategy. Significantly, we have shown a differential effect of the compound, which is dependent on genetic background of cells that could also influence treatment choice. In addition, to our knowledge, this is the first study showing anti-growth activity of the Pd complexes against CSCs and it thereby warrants further investigation as a chemotherapeutic for prostate cancer.

## Methods

### Culture of Cell Lines

In this study, six different prostate cell lines (PNT1A, PNT2-C2, BPH-1, PC-3, LNCaP, P4E6) were used (Table 1). These cell lines encompass the spectrum of cellular differentiation status (basal, intermediate and luminal phenotypes), as well as the spectrum of normal, early cancer and late cancer. BPH-1 is derived from benign prostate hyperplasia (BPH), while PNT2-C2 and PNT1A are derived from normal prostate. PC-3, LNCaP and P4E6 are cancer cell lines. LNCaP, PNT2-C2, PNT1A were grown in RPMI medium with 10% FCS (foetal calf serum); PC-3 was grown in Ham’s F-12 medium with 7% FCS; BPH-1 was grown in RPMI medium with 5% FCS; P4E6 was grown in KSFM (Keratinocyte serum free medium) with 2% FCS. No antibiotics were used in any media. The cells were incubated at 37°C in a humidified atmosphere containing 5% CO_2_.

**Table 1:** Cell lines.

Cell line	Diagnosis	Source/Reference
PNT1A	Normal prostate epithelium immortalized with SV40	Kind gift to the lab of Norman Maitland from P. Berthon Currently available from Health Protection Agency Culture Collections.
PNT2-C2	Normal prostate epithelium immortalized with SV40	Obtained from ECACC (no longer available from ECACC)
BPH-1	Primary epithelial culture from benign prostatic hyperplasiaimmortalized with SV40	Obtained by Norman Maitland, with kind permission from Simon Hayward [49]. Not commercially available.
P4E6	Epithelial culture established from well-differentiated prostate cancer/E6gene from human papillomavirus introduced by retroviral insertion	Derived in York [50]. Currently available from Health Protection Agency Culture Collections.
PC-3	Prostate adenocarcinoma/bone metastasis	ATCC
LNCaP	Prostate carcinoma/lymph node metastasis	ATCC

### Culture of Primary Prostate Epithelial Cells

Primary prostate epithelial cells were isolated from human tissue samples. The samples were collected with ethical permission from York District Hospital (York, UK) and Castle Hill Hospital (Cottingham, UK). Benign prostatic hyperplasia (BPH) and prostate cancer samples were obtained from TURP (transurethral resection of the prostate), radical prostatectomy (laparoscopic and open) and cystectomy operations. All patients gave written consent for their tissue to be used for research and all patient samples were anonymised. Permission was approved by the Local Research Ethical Committees, associated with York District Hospital and Castle Hill Hospital. Permission was administered by the Yorkshire and Humber Research Ethics Committee.

Prostate epithelial cells were cultured as previously described [26] in stem cell media (SCM) consisting of keratinocyte serum-free media (KSFM) with bovine pituitary extract (BPE) and epidermal growth factor (EGF), glutamine, stem cell factor (SCF), granulocyte macrophage colony stimulating factor (GM-CSF), leukaemia inhibitory factor (LIF) and cholera toxin. Cells were grown with STO feeder cells (irradiated at 60 Gy) on collagen I-coated plates. The detailed information of the primary cells used is given in Table 2.

**Table 2:** Primary epithelial cells.

Sample	Passage	Operation	Diagnosis
01409	6	C	benign
03108	6	T	benign
01608	2+3	T	benign
08109	4	T	benign
00409	4	T	benign
07611	2	T	benign
22612	3	T	benign
05908	2	T	benign
07011la	3	R	Cancer on hormones Gleason 7
07011lb	3	R	Cancer on hormones Gleason 7
05411rb	5	R	Cancer Gleason 7
07311ra	3	R	Cancer Gleason 7
06711rb	6	R	Cancer Gleason 6
06211rb	4	R	Cancer Gleason 7
06611lb	5	R	Cancer Gleason 7
04811rb	5	R	Cancer Gleason 6
06411ra	3	R	Cancer Gleason 7
06411lb	3	R	Cancer Gleason 7
25212ra	3	R	Cancer Gleason 7
23912ra	6	R	Cancer Gleason 9
22412	2/3	chT	Cancer Gleason 7
22112	4	R	Cancer Gleason 7
22012ra	5	R	Cancer Gleason 7
07311la	7	R	Cancer Gleason 7
16312	5	chT	Cancer Gleason 8
22912	2	chT	Cancer Gleason 8/9
14912	3	chT	Cancer Gleason 9

C = Cystectomy/T = Transurethral resection of the prostate/R = Radical Prostatectomy/chT = channel TURP.

### Isolation of Cancer Stem Cells, Transit Amplifying Cells and Committed Basal Cells

Epithelial cells from human tumour or BPH materials were cultured for several weeks and the cells treated at very low passages. The cultured basal cell population were trypsinized, resuspended in SCM and then plated on BSA-blocked collagen I-coated plates. After 30 min, cells that did not attach to the substratum were collected, consisting of the committed basal cells (CBs), which are α2β1integrin^lo^. The cells that attached to substratum were trypsinised, resuspended in MACs buffer and incubated with CD133-microbeads (Cat no. 130-050-801, Miltenyi Biotec Inc., Auburn, CA, USA). MACs MS columns (Cat no. 130-042-201, Miltenyi Biotec Inc., Auburn, CA, USA) were used to select the CD133^+^ and CD133^−^ cells. Finally, the three cell populations were obtained: stem cells (SCs) - α2β1integrin^hi^/CD133^+^, transit-amplifying cells (TAs) - α2β1integrin^hi/^CD133^−^ and committed basal cells (CBs) - α2β1integrin^lo^.

### Chemicals

**Figure 1:**
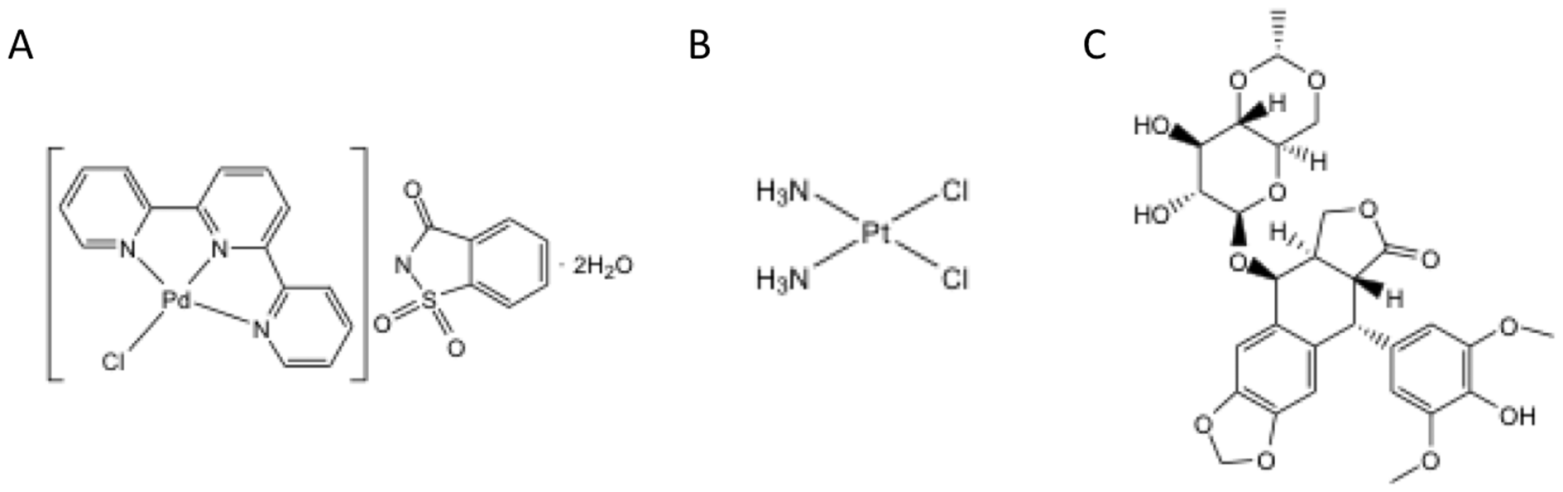
Chemical structures. (A) Palladium complex [PdCl(terpy)](sac)·2 H_2_O, (sac = saccharinate, and terpy = 2,2′:6′,2″-terpyridine). M = Palladium(II) (B) Cisplatin (C) Etoposide.

The palladium (Pd) complex was synthesized in the Chemistry Department of the Science and Art Faculty of Uludag University. The synthesis, characterization and crystal structure of the palladium(II) complex has been reported elsewhere [23]. [PdCl(terpy)](sac)⋅2 H2O was synthesized by the direct addition of an equimolar amount of sac ions to [Pd(terpy)Cl]Cl⋅2 H2O in solution in high yield. The orange crystals of the compound were obtained and its molecular structure was confirmed by X-ray diffraction. The chemical structure is shown in Figure 1A. Stock and final concentrations of the Pd complex were prepared in the appropriate culture medium. The Pd complex was used at concentrations ranging from 0.39 to 100 µM. Cisplatin (sc200896, Santa Cruz Biotechnology, Santa Cruz, CA, USA) (Figure 1B) and Etoposide (E1383, Sigma-Aldrich, Saint Louis, MO, USA) (Figure 1C), were used as positive controls for cytotoxic activity at doses of 25 µM and 12 µM or 6 µM, respectively.

### MTS Assay

This assay was performed for the initial screening of the effect of the Pd complex on the cell lines and the whole cell population of primary cultures. The CellTiter 96® Aqueous One Solution Cell Proliferation Assay kit (G3580, Promega, Madison, WI, USA) was used and the manufacturer’s instructions were followed. Briefly, after treating cells that were seeded at a density of 5,000 cells per well in a 96-well plate in triplicate for a desired period (24 h, 48 h, 72 h), 20 µL of reagent was added to each well. Following 2.5 h incubation at 37°C, the absorbance was read at 485 nm using a plate reader (PolarStar Optima, BMG Labtech, UK). Percent viability was calculated using the formula (% Viability = [(Sample Absorbance/Control Absorbance)]×100.

### ATP Assay

This assay was employed for the bioluminescent determination of the adenosine 5′-triphosphate (ATP) released from fractionated living cells (cancer stem cells, transit amplifying cells and committed basal cells). As ATP is rapidly degraded in dead cells, high intracellular levels provide a selective assay for living cells. The ATP-bioluminescent somatic cell assay kit (FLASC, Sigma-Aldrich, Saint Louis, MO, USA) was used with protocol modifications. Briefly, 50–500 cells per well were seeded in a collagen-coated 96 well plate in triplicate. After treating cells for 72 h, 150 µL of medium was removed from each well. 50 µL of cell extraction reagent was added into each original well. Following 20 min incubation at RT, 50 µL of cell extract was transferred to a white 96-well plate. Finally, 50 µL of ATP assay mix solution was added into the wells and luminescence was read using a plate reader (PolarStar Optima, BMG Labtech, UK). Percent viability was calculated using the formula (% Viability = [(Sample RLU/Control RLU)]x100 where RLU refers to relative light units.

### Immunofluorescence

γH2AX staining: Cells were seeded onto 8-well collagen I-coated chamber slides. Briefly, following 48 h treatment with the Pd complex or etoposide, cells were washed with PBS and fixed with 2% paraformaldehyde in PBS with 0.2% Triton X-100, pH 8.2 for 20 min, and then permeabilised with 0.5% NP40 in PBS for 20 min at RT followed by three washes with PBS. After blocking of non-specific binding with 2% BSA in PBS with 1% goat serum for 1 hour at RT, primary antibody (anti-phospho-histone H2A.X (Ser139), clone JBW301, Millipore, Cat no. 05–636) at 1∶1000 dilution was added in 3% BSA in PBS at 4°C overnight followed by three washes in 0.5% BSA in PBS with 0.175% Tween 20. Following incubation with secondary antibody (Goat anti-mouse Alexa Fluor 568, Invitrogen, Cat no. A-11004) at 1∶1000 dilution for 45 min in 3% BSA in PBS and three more washes in the same washing buffer, the slides were mounted using Vectashield with DAPI (Vector Laboratories, Cat no. H-1200). LC3-B staining: Cells were treated as above then fixed with 4% paraformaldehyde, followed by an incubation in 0.3% Triton X-100. After blocking with 10% normal goat serum, cells were incubated in anti-LC3B 1∶200 (Ab51520, abcam) diluted in 0.1% Triton X-100 in PBS. Secondary antibody was Alexa Fluor 568 goat anti-rabbit IgG 1∶1000 (Invitrogen A11011).

### Flow Cytometry

Following drug treatment, floating cells in the media were pooled with adherent cells, which were collected by trypsinisation. Following centrifugation cells were resuspended in 0.5 ml PBS. Cells were fixed in 2 ml ice cold 70% ethanol, which was added in a dropwise fashion while vortexing. Cells were incubated on ice for 30 min then washed in 5 ml PBS and resuspended in 0.4 ml PBS. Following storage at 4°C overnight, 50 µl of RNAse (1 mg/ml) and 50 µl of propidium iodide (1 mg/ml) were added to the cells. Following incubation at 37°C for 30 min the cells were analysed for 2N and 4N DNA content on a flow cytometer (Cyan ADP Analyser, Beckman Coulter).

### Western Blotting

Following drug treatment, cell lysates were harvested using Cytobuster Protein Extraction Reagent (71009, EMD Millipore, Darmstadt, Germany) with protease inhibitors (cOmplete, EDTA-free Protease Inhibitor Cocktail Tablets, Roche Applied Science, UK). 20 µg of protein extract were loaded on 12% SDS-PAGE gels and wet-transferred to a PVDF membrane. Antibodies used include: monoclonal anti-β-actin 1∶5000 (A5316, Sigma-Aldrich), anti-LC3B 1∶3000 (Ab51520, abcam), cleaved caspase-3 (Asp175) 1∶1000 (9661S, Cell Signaling Technology) and secondary antibodies were Rabbit anti-mouse-HRP 1∶10000 (P0260, Dako) and anti-rabbit IgG HRP-linked 1∶5000 (Cell Signaling Technology Inc. 7074S). Kaleidoscope protein marker was run on each gel (161-0324, Bio-Rad).

### Statistics and Calculations

MTS and ATP assays were performed in triplicate and data presented as the mean +/− standard deviation. IC_50_ values (Table 3 and Table 4) were calculated from graphs of transformed data following application of the nonlinear regression (curve fit) that represents the log(inhibitor) ‘v’ normalized response (GraphPad Prism software) (Supplementary Figures S1 and S2). For significance calculations, the Wilcoxon rank sum test was used (Sigmaplot). Flow cytometry analysis was carried out in triplicate and results are presented as an average with error bars indicating the standard error.

**Table 3:** IC_50_ values of the Pd complex in cell lines.

Cell lines 24 h	IC_50_ (µM)	Cell lines 48 h	IC_50_ (µM)	Cell lines 72 h	IC_50_ (µM)
PNT1A	27.97	PNT1A	1.258	PNT1A	0.1064
PNT2-C2	1.732	PNT2-C2	17.17	PNT2-C2	0.9033
BPH-1	17.62	BPH-1	1.881	BPH-1	0.1399
***AVERAGE***	**15.774**	***AVERAGE***	**6.769666667**	***AVERAGE***	**0.3832**
P4E6	21.80	P4E6	9.660	P4E6	4.372
PC-3	98.91	PC-3	49.15	PC-3	26.79
LNCaP	105.5	LNCaP	11.90	LNCaP	3.433
***AVERAGE***	***75.40333333***	***AVERAGE***	***23.57***	***AVERAGE***	***11.53166667***

**Table 4:** IC_50_ values of the Pd complex in primary cells.

BPH primary cells	IC_50_ (µM) New
7611	6.974
1608	5.969
3108	7.326
8109	14.43
***AVERAGE***	***8.67475***
**PCa Primary cells (Gl7)**	**IC_50_ (µM) New**
06411ra	3.778
22412	6.303
06411lb	5.196
07011la	5.357
07011lb	6.025
07311ra	5.371
06211rb	11.06
06711rb	8.241
06611lb	13.12
***AVERAGE***	***7.16122***
**PCa Primary cells (Gl8/9)**	**IC_50_ (µM)**
16312	70.41
22912	46.68
14912	64.07
***AVERAGE***	**60.38666667**

## Results

### Effect of Pd Complex on Cell Lines

**Figure 2:**
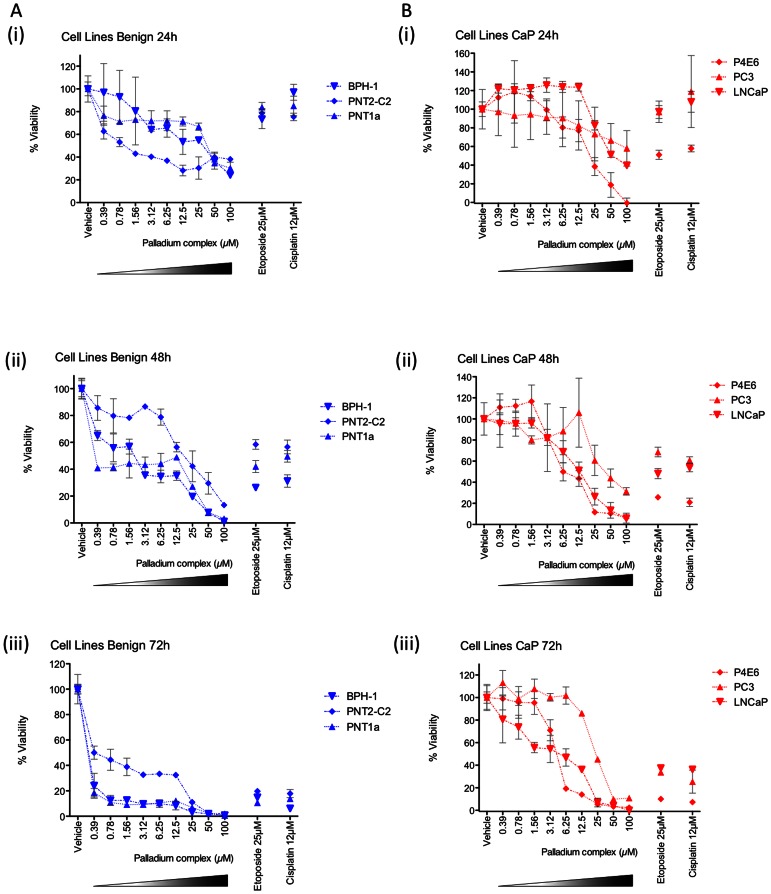
Anti-growth effect of the Pd complex on cell lines. Anti-growth effect was measured by the MTS assay at 24 h, 48 h and 72 h. PNT1A, PNT2-C2, and BPH-1 have either normal tissue or benign prostatic hyperplasia tissue origin (Ai–iii), while PC-3, LNCaP and P4E6 cell lines have malignant origin (Bi–iii). IC_50s_ are presented in Table 3. Transformed graphs are presented in Supplementary Figure S1.

The anti-growth effect of the Pd complex was tested against six different cell lines at three different time points, 24 h, 48 h and 72 h (Figure 2). The Pd complex inhibited the growth of all cell lines almost completely at 100 µM concentration at 72 h. A comparison was made to etoposide (25 µM) and to cisplatin (12 µM), used as known cytotoxic agents. At 72 h, the lowest IC_50_ value, 0.1399 µM, was for the BPH-1 cell line, with PNT1A cells having a similarly low IC_50_ of 0.1064 µM, while PNT2-C2 cells were more resistant, with an IC_50_ value of 0.9033 µM (Table 3). The well differentiated early stage prostate cancer cell line P4E6, and LNCaP, which is from a lymph node metastasis with the most luminal phenotype (androgen-positive) had IC_50s_ of 4.372 µM and 3.433 µM, respectively, whereas the cancer cell line from a bone metastasis, PC-3, had an IC_50_ value of 26.79 µM. There was a less dramatic effect on PNT2-C2 cells compared to the other normal and benign cell lines (Figure 2A(iii)). However, the cancer cell line from the bone metastasis is least susceptible to the drug, with a significantly higher IC_50_ (Figure 2B(iii)). Overall, the Pd complex successfully reduced viability of all cell lines tested with some variation in response.

### Effect of Pd Complex on Primary Cultures from Benign and Malignant Samples

**Figure 3:**
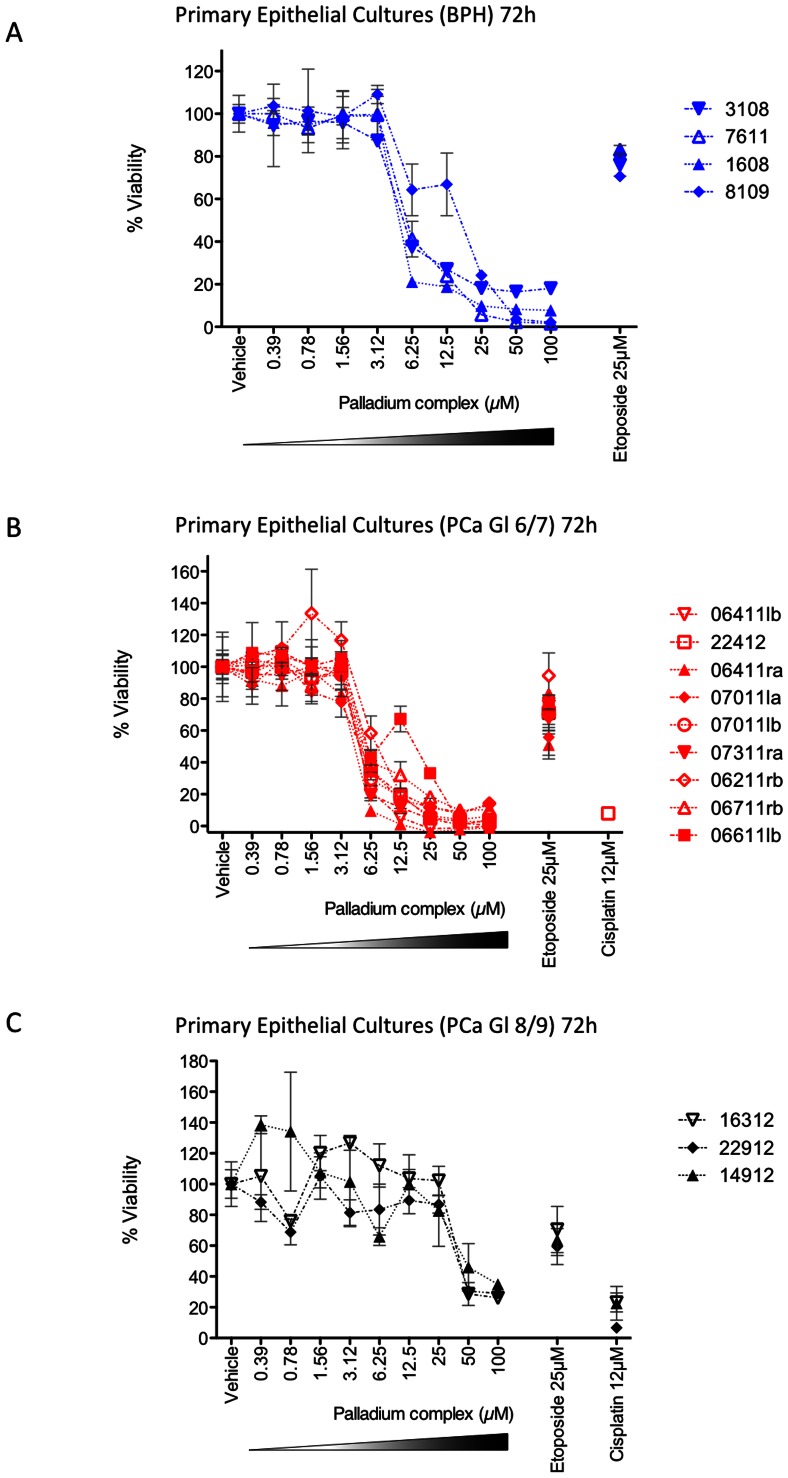
Anti-growth effect of the Pd complex on primary cultures. Anti-growth effect was measured by the MTS assay at 72 h using cells derived from patients with (A) benign prostate hyperplasia, (B) prostate carcinoma with Gleason grade 6/7 and (C) prostate carcinoma with Gleason grade 8/9. IC_50s_ are presented in Table 4. Transformed graphs are presented in Supplementary Figure S2.

The most complete dose response was observed at the 72 h time-point, and so this time point was used to explore the anti-growth effect of the Pd complex on primary cultures from patient tissue to assess a model closer to the disease state. The Pd complex was tested on primary cultures derived from patients with benign prostate hyperplasia (BPH, n = 4 from four patients) (Figure 3A), prostate carcinoma with low Gleason grades (6/7) (n = 9 from seven patients) (Figure 3B) and with high Gleason grades (8/9) (n = 3 from three patients) (Figure 3C). The dose response curve was strikingly similar between BPH and Gleason 6/7 samples, with concentrations higher than 6.25 having a significant anti-growth effect on all samples. Compared to etoposide, the Pd complex at the same concentration (25 µM) was found to significantly reduce cell viability at least 5.6-fold in benign samples (P = 0.029) and 10.66-fold in malignant samples (P = <0.001) (median values used to calculate fold difference). Significantly, the Pd complex had a less pronounced effect in high Gleason grade (8/9) prostate cancer (Figure 3C). For the benign and Gleason 6/7 cancer cultures the average IC_50_ was 8.67 µM and 7.16 µM respectively, while for the high Gleason grade cancers (8/9) the IC_50_ was 60.39 µM (Table 4), indicating that these cultures are more resistant, or less susceptible to the complex. Considering the need for new drugs to treat high Gleason grade tumours that are often radiorecurrent and hormone-resistant, this is a significant observation regarding these samples, and one that could have been missed if using only cell lines.

### Effect of Pd Complex on Cancer Stem Cells from Primary Epithelial Cultures

**Figure 4:**
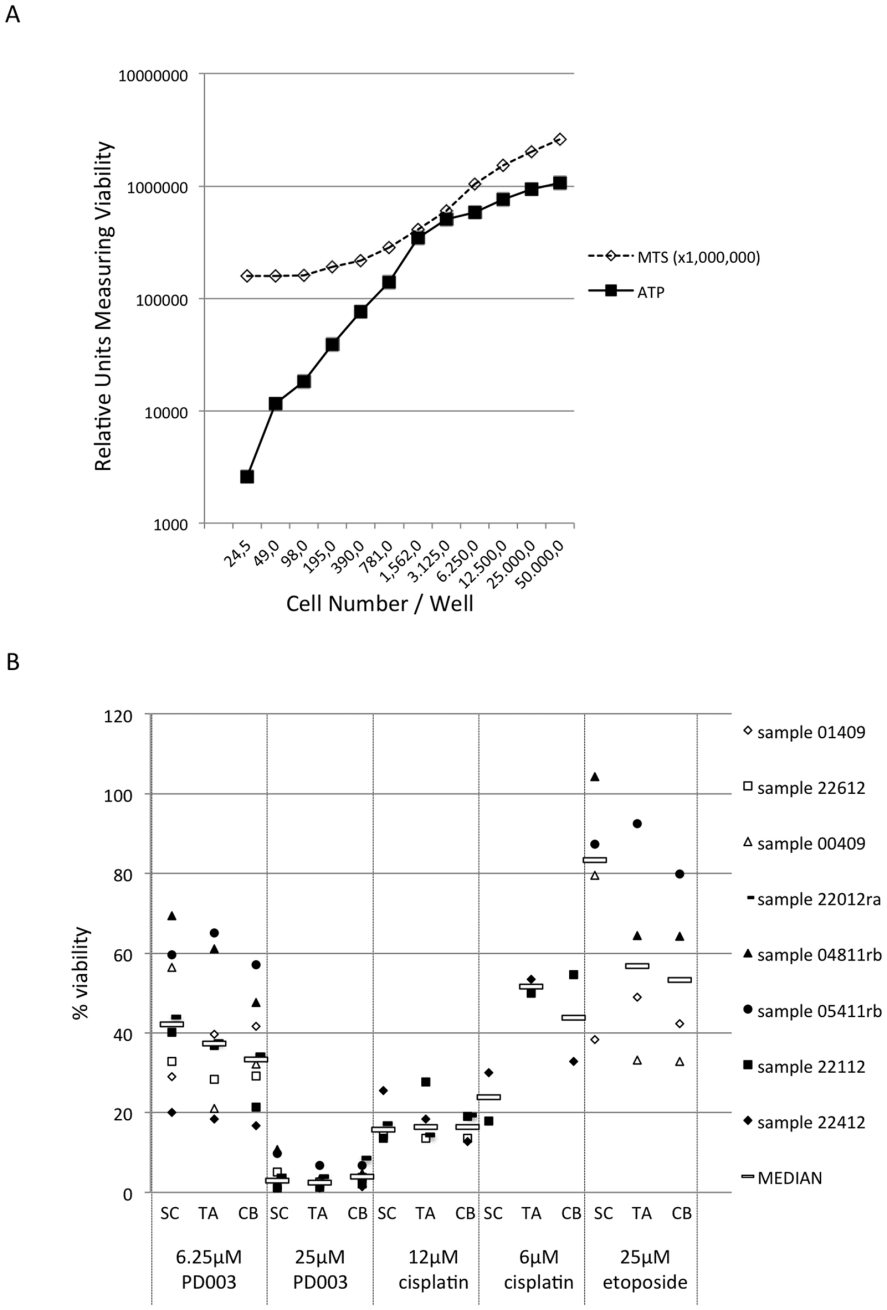
Anti-growth effect of the Pd complex on cancer stem cells (CSC). (A) MTS assay and ATP assay were compared to assess anti-growth effect using small cells numbers. (B) Anti-growth effect was measured by the ATP assay using SCs, TA cells and CB cells derived from three patients with benign prostate hyperplasia (white-filled shapes) and five patients with prostate carcinoma (black-filled shapes). White bar represents the median value of all the samples.

Prostate tumours are heterogeneous, and so the anti-growth effects of the Pd complex specifically on benign and malignant stem cells (SCs) were explored. SCs were isolated from primary cultures derived from three benign and five prostate carcinoma (Gl6/7) patient samples. In addition to SCs, TA cells, and CB cells were also isolated. The SCs are a rare population of cells, and as such the anti-growth effect was measured by the ATP assay, since it significantly more sensitive than the MTS assay and can accurately measure low cell numbers (Figure 4A). The Pd complex was tested at two different doses (6.25 and 25 µM) on the basis of previous experiments where 6.25 µM was the lowest concentration inducing a significant anti-growth effect and 25 µM caused a dramatic reduction in cell viability (80%–100% in BPH and Gleason 6/7 cancers). It is clearly shown that 25 µM Pd complex was significantly more cytotoxic in stem cells, compared to 25 µM etoposide (Figure 4B). (Using a Wilcoxon rank sum test to measure the effect of 25 µM etoposide versus 25 µM PD003, the latter is significantly more cytotoxic with a P value = 0.004 in all three tests, comparing each population separately). The 6.25 µM Pd complex resulted in an anti-growth effect that was less than 25 µM Pd complex but still more cytotoxic than 25 µM etoposide. (Using a Wilcoxon rank sum test to compare 6.25 µM Pd complex versus 25 µM Pd, there is a significant difference in cytoxicity with a P value = <0.001 in all three tests, comparing each population separately). Cisplatin also appeared to be significantly cytotoxic to all cell populations; 6 µM of cisplatin was equivalently cytotoxic to 6 µM Pd complex (P values showed no significant difference SC‘v’SC = 0.073, TA‘v’TA = 0.4, CB‘v’CB = 0.533). SCs appeared to have increased viability compared to TA and CB cells following etoposide treatment (although this was not statistically significant), which was not the case following Pd complex treatment.

### DNA-damaging Effect of Pd Complex

**Figure 5:**
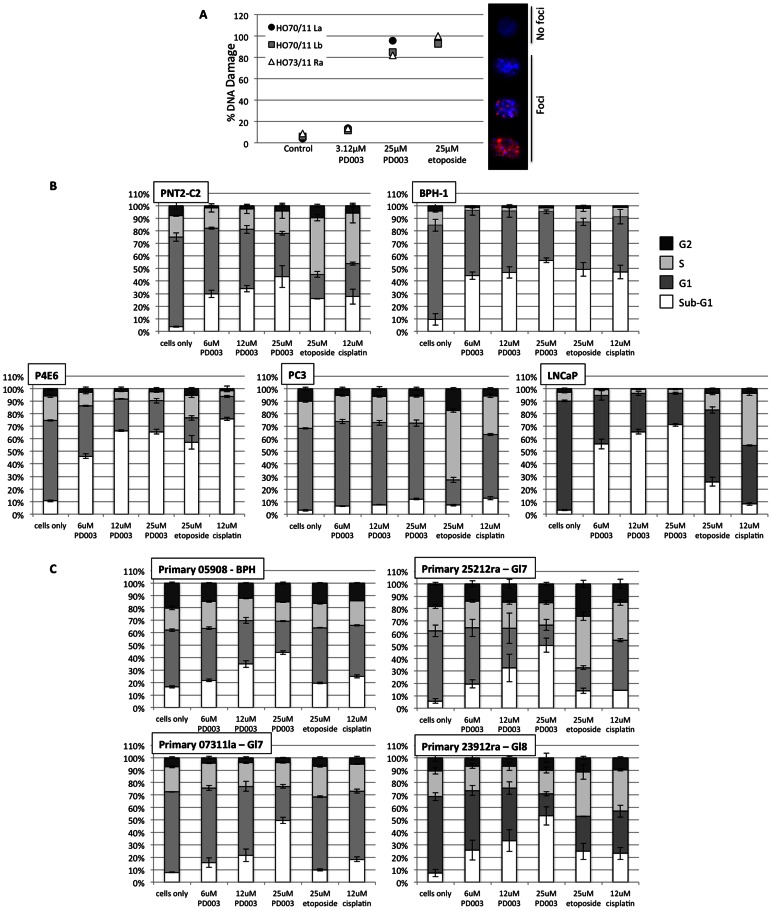
DNA-damaging effect and effect on Cell Cycle Status of Pd complex. (A) Primary cultures isolated from two patients with prostate carcinoma were assessed. Cells were stained and scored for nuclear foci indicative of DNA damage Representative examples of cells negative and positive for nuclear foci are shown. (B) Normal (PNT2-C2) and benign (BPH-1) cell lines, three cancer cell lines (P4E6, PC-3 and LNCaP) and (C) four primary cultures derived from patients with prostate carcinoma were treated with three concentrations of palladium complex or etoposide or cisplatin as control treatments. Cell cycle phase was measured using propidium iodide staining and flow cytometry analysis.

Since the mechanism of action of the Pd complex has not been fully characterized, the DNA-damaging effect was assessed. 10,000 cells per well in 8-well chamber slides were treated for 48 h with Pd complex. Nuclei with evidence of γH2AX nuclear foci, indicative of DNA damage, were counted on 10 randomly chosen fields at the highest (63x) magnification and the mean number of positively-stained nuclei was calculated. At least 100 cells per well were counted. Both etoposide and Pd complex at the same dose yielded similar levels of DNA damage (Figure 5A). 3.12 µM of the Pd complex did not induce a significant level of DNA damage.

### Effect of Pd Complex on the Cell Cycle

Following on from the observation that the Pd complex caused DNA damage, we explored its effect on cell cycle status, since DNA damage can lead to activation of cell cycle checkpoints and cell death (Figure 5B–C, Supplementary Figure S3). Etoposide caused an S phase arrest in PNT2-C2, PC3 and LNCaP cell lines and also in primary prostate epithelial cells (measured at 48 h post-treatment), which is not unexpected since etoposide treatment leads to DNA damage in the S phase of the cell cycle [27]. Following treatment with the Pd complex, the cell lines showed an increase in cells with sub-G1 DNA content, indicative of cell death in all cases, except PC3 cells where there was almost no change. Of the other cell lines, the PNT2-C2 cells were the least susceptible. Generally, at lower concentrations (6 µM and 12 µM), the Pd complex showed similar levels of cell death to the cisplatin and etoposide controls. In the normal cells (BPH-1, PNT2-C2), the increase in cells with sub-G1 DNA content was accompanied by a reduction in the S and G2 peaks, indicative of either a G1 arrest followed by cell death, or a cell replication failure preceding cell death. In primary cells, treatment with the Pd complex gave a clear dose response showing increase in sub-G1 content, and also induced an increase in cells with more than 4N DNA content, potentially indicative of induction of aneuploidy. Similarly to etoposide, cisplatin caused an S phase arrest in PNT2-C2 cells, LNCaPs and PC3 cells as well as primary cells. Overall, it appears that the Pd complex had a different effect on the cell cycle status than either etoposide or cisplatin.

### Induction of Apoptosis and Autophagy by the Pd Complex

**Figure 6:**
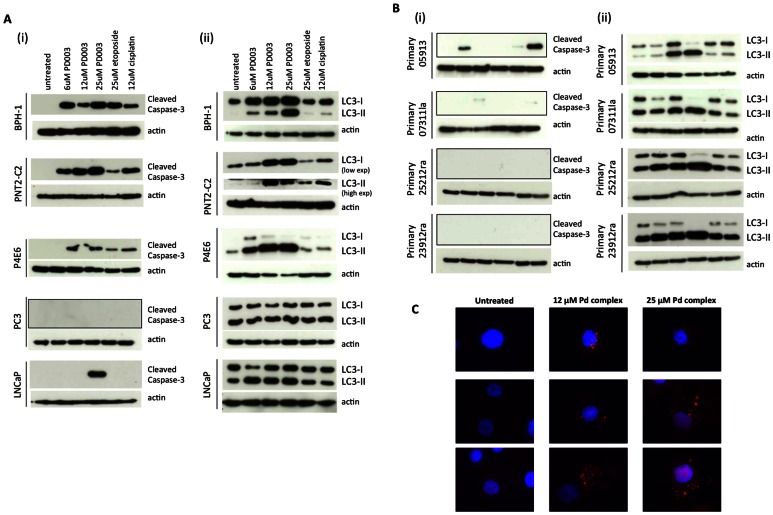
Expression of apoptosis and autophagy-related proteins following treatment with Pd complex. Normal (PNT2-C2) and benign (BPH-1) cell lines, three cancer cell lines (P4E6, PC-3 and LNCaP) (A) and four primary cultures (B) one derived from patient with BPH and three derived from patients with prostate carcinoma were treated with three concentrations of palladium complex or etoposide or cisplatin as control treatments. Lysates were harvested and Western blotting was carried out staining for cleaved caspase-3 indicative of apoptosis induction or LC3B protein, indicative of autophagy. (C) Images of primary cells (sample 23912) treated with Pd complex and stained with LC3-B antibody. Shown are three example images of untreated cells and cells treated with 12 µM and 25 µM Pd complex. Autophagosome vesicles are clearly visible in red.

The indication of reduced cell growth resulting from the cell proliferation assays, along with the increase in sub-G1 content of the treated cell populations, led to the investigation of cleaved caspase-3 activity to assess induction of apoptosis (Figure 6A(i), Supplementary Figure S3). There was a clear induction of cleaved caspase-3 in BPH-1, PNT2-C2 and P4E6 cells when treated with Pd complex, etoposide and cisplatin. However induction of cleaved caspase-3 in LNCaP cells was only observed after treatment with 25 µM Pd complex, and there was no evidence of cleaved caspase-3 in PC3 cells that present as a very resistant cell line. This also correlated with the much lower percentage of PC3 cells with a sub-G1 DNA content. More significantly, there was no evidence of cleaved caspase-3 in two primary samples (Figure 6B(i)), and only a positive result with the BPH sample at a low dose. Since the sub-G1 content increases in primary cells in response to Pd complex but with no corresponding caspase activity, this may mean that the apoptotic kinetics differ between the primary cells and cell lines or that the sub-G1 content in the primary cells could be attributed to necrosis. To investigate the mechanism of death in the cell lines and indeed the absence of apoptosis in the primary cells, levels of LC3-I and LC3-II were measured, to assess autophagy. The ratio of LC3-II to LC3-1 used to be the standard measurement of autophagy, however it is now accepted that levels of LC3-II alone should be assessed relative to a typical control such as actin [28], [29]. There was a clear increase in the expression of LC3-II in BPH-1, PNT2-C2 and P4E6 cells following increasing doses of Pd complex (Figure 6A(ii). Treatment with etoposide or cisplatin did not significantly change levels of LC3-I and LC3-II. The levels of LC3-I and LC3-II in LNCaP and PC3 cells did not change dramatically or in a dose-dependent fashion with any treatment. In all primary cells there was a clear increase in LC3-II, the modified version of LC3-I that is present on the autophagosomes and indicative of autophagy [29], with increasing Pd complex treatment (Figure 6B(ii)). This was also observed using immunofluorescence and autophagosomes were observed in primary cells following treatment with Pd complex (Figure 6C). Again, there was no significant change in LC3-I or LC3-II levels following etoposide or cisplatin treatment. This provided further clear evidence that the mechanism of action of the Pd complex is different to etoposide or cisplatin, and indeed different between the cell types studied.

## Discussion

Recurrent prostate cancer eventually results in the death of the patient due to resistance to chemotherapy and ineffective chemotherapy, almost inevitably within 2 years from the failure of hormone treatment [4]. Therefore, more efficient drugs/approaches are required. In this study, we investigated the anti-growth effect of a novel palladium complex, which is a growing area in anti-cancer drug development. *In vitro* studies on different kinds of palladium complexes recently synthesized by both our group and others have produced promising results [19], [20], [30]. In addition to *in vitro* studies, our *in vivo* study on breast cancer cell lines also resulted in considerable cell death by inducing apoptosis via cell death receptors, as well as inhibition of angiogenesis [25].

In this study, we have found that the Pd complex had a significant growth-inhibiting activity against both prostate cancer cell lines and cell lines derived from normal and benign prostate. IC_50_ values have ranged from 0.1064 µM to 26.79 µM (72 h), depending on the cell line. In the literature, IC_50_ values of palladium compounds also have a broad range. In the study of Nadeem et al [18] it varied from 5.80 to >100 µM. The reason for this broad range may be related to the nature of ligands attached to the palladium metal core. In another study, in which different cell lines from different cancer types (central nervous system, colon, breast, leukemia, and prostate) were used, the Pd complexes resulted in broad range (13 to >100 µM) of IC_50_ values depending on both cell types and the mono- and dinuclear Pd complexes [31]. Taken together, the great variability in IC_50_ values seems to depend on both cell type and the ligand attached. In the study of Mukherjee et al using palladium complexes, prostate cancer (PC-3) cells died by apoptosis following cell cycle arrest at G2/M phase [20]. Ultimately, it would be hoped that a new drug preferentially killed cancer cells over normal cells. Although it was first encouraging that one of the normal cell lines, PNT2-C2, appeared to be less susceptible to the Pd complex than the other normal (PNT1A) and benign (BPH-1) cell lines, once compared to the cancer cell lines it became apparent that the cancer cell lines are overall less susceptible to the Pd complex with average IC_50_ values at 72 h being 11.53 µM, whereas the normal cell lines had an average of 0.38 µM. Therefore, more drug is required to reduce viability of the cancer cells. This is disappointing but unfortunately not surprising, and new approaches to modify the compound in order to target it to the tumour while sparing the normal tissue would be desirable. Significantly, this study shows the importance of using a panel of cell lines, and not just one ‘normal’ and one ‘cancer’ cell line. There is variability between the normal versus cancers, just as there is variability between the different normal cell lines and different cancer cell lines.

Cell lines are very commonly used for initial high throughput screening of cytotoxic anti-cancer compounds. However, it is physiologically more relevant to use primary cultures to obtain results that are closer to the patient. Therefore, in addition to the many cell lines used in this study, we studied the anti-growth effect of the Pd complex on primary cultures from patient tumour samples (Gleason grade 7). We found that the Pd complex had a powerful growth-inhibiting effect on these primary cancer cells. Most of the IC_50_ values had quite a narrow range of around 3.778 to 13.12 µM depending on the patient from whom the cells were isolated. This was comparable to the cancer cell line IC_50_ values. Interestingly, when the Pd complex was tested against the cells isolated from benign prostatic hyperplasia patients the results were quite similar to those found in the malignant samples with IC_50_ values ranging from 5.969 µM to 14.43 µM. Therefore, once again the Pd complex did not preferentially kill cancer cells, but importantly did not preferentially kill benign primary cells (unlike the normal cell lines). However, more significantly, when the drug was tested against cultures from high Gleason grade tumours (Gleason 8/9) the IC_50_ range for these samples was 46.68–70.41 µM. Therefore, around ten times higher concentration of the drug is required (using median values) to reduce the viability of these aggressive cancers compared to the lower grade cancers. This is a statistically significant difference, P = 0.016. This is the first study testing this novel compound on primary epithelial cell cultures of prostate and clearly highlights the utility of both cell lines and primary cells when assessing a new drug.

There is now increasing evidence that cancer stem cells are responsible for the recurrence of disease, due to their resistance to current chemotherapy [32], [33], [34]. Therefore, we investigated the effect of the Pd complex on cancer stem cells isolated from malignant samples and stem cells isolated from benign prostate hyperplasia samples. In addition to CSCs, TA and CB cells from the same cultures were also isolated and studied. We found that the Pd complex had much more potent cytotoxic activity than etoposide at the same concentration (25 µM) but had comparable toxicity to cisplatin at the 6 µM range. This is also the first report in the literature, to our knowledge, on the effect of Pd complexes on cancer stem cells. Eradication of cancer stem cells is an aim of most novel strategies, but with the potential for these cells to display increased resistance, it is likely that they may need more rigorous treatment. In fact, in our study we also used the concentration of 50 µM of the complex, which killed all cells (data not shown). However, this concentration may be too toxic to the other cells of the body, and thus not be tolerated by patients. Indeed, although we observed significant anti-growth effects of cancer cells, this Pd complex also efficiently killed normal and benign cells. The overall toxicity of the drug will be an important future consideration.

To investigate the mechanism of cell death and the effect of the Pd complex on the cell cycle, we first studied the DNA-damaging effect of the Pd complex. Platinum compounds are known to induce DNA adducts, which block replication and transcription resulting in DNA damage and cell death [35]. Interestingly, we found that both Pd complex and etoposide caused the same level of DNA damage, measured as γH2AX foci (indicative of double-strand breaks) although the Pd complex resulted in a more powerful cytotoxic effect than etoposide. This implies that some of the DNA damage resulting from etoposide treatment may be repairable, but the damage caused by the Pd complex may not, and is therefore more lethal. Future studies will develop this area and will investigate the types of DNA damage occurring as well as the active DNA repair mechanisms. It is known that there can be resistance to platinum-based drugs and that this can be related to the DNA repair mechanisms in the cell [35]. It will therefore be interesting to determine if cells also acquire resistance to palladium complexes, and whether this is through similar mechanisms, or indeed if they are less likely to acquire resistance to these compounds. Following increase of the sub-G1 population in the cell cycle analysis, induction of apoptosis was confirmed with Western blotting of cleaved caspase-3. Interestingly apoptosis was induced in all of the cell lines (only at the highest Pd complex concentration for LNCaP), except PC3. No apoptosis was induced in the primary cancer cells.

In both cell lines and primary cells, autophagy was measured by assessing levels of LC3-I and LC3-II. Increased levels of LC3-II protein typically hints at induction of autophagy, although future studies would have to incorporate autophagic flux to elucidate the complete response [28], [36]. Increased levels of LC3-I may occur prior to an increase in LC3-II or could indicate that there is a block in autophagy at an early stage. Autophagy is a dichotomy in cancer because in some circumstances it can be a cell-protective survival mechanism responding to hypoxia, nutrient deprivation or stress, whilst in other circumstances it can be a prelude to autophagy-induced caspase-independent cell death [37], [38]. Indeed, autophagy and apoptosis are activated by similar stimuli but can be mutually exclusive or simultaneous [37]. In terms of using autophagy as a treatment strategy in cancer, this has been approached in two ways. In CML, the combination of a TK inhibitor with an autophagy inhibitor increased cytotoxicity [39], [40]. Secondly, use of temolozide, a pro-autophagic drug, in glioblastoma in combination with an mTOR inhibitor induced autophagy and cell death [41]. Therefore, it is imperative to understand the biology of the cancer cells under treatment before deciding on the best strategy. Indeed, within this dichotomous role, it seems that autophagy is less active in early stage cancers with its cell-protective role, only coming to the fore in later stage cancers [41], [42], [43]. In terms of prostate cancer, there have already been studies indicating that autophagy protects against hormone ablation therapy, and combining androgen deprivation with autophagy inhibition led to synergistic cell death suggesting a new potential strategy to overcome hormone therapy resistance [44]. There are also clinical trials underway combining docetaxel (standard chemotherapy for prostate cancer) with hydroxyquinone autophagy inhibitor [45]. From our results, the presence or absence of PTEN may contribute to the outcome of these strategies. In PC3 and LNCaP cells the palladium compound did not induce autophagy and only induced apoptosis at high concentrations (LNCaP) or not at all. Both cell lines contain inactive PTEN [46] and since mTOR is a key negative regulator of autophagy, the absence of PTEN, a negative regulator of mTOR, could result in lack of induction of autophagy. In prostate cancers around 40% of patients lack PTEN activity, a proportion that increases in castration-resistant prostate cancer [47], [48]. The other cell lines tested (BPH-1, PNT2-C2 and P4E6) are PTEN-positive and were susceptible to the drug. Thus, there may be differential efficacy of this combination strategy between patients, depending on the genetic background of the tumour.

In conclusion, the Pd complex had a considerable anti-growth effect on most prostate cancer cell lines and primary cultures. Importantly, it also successfully inhibited the viability of cancer stem cells, implying that this Pd complex may be used for the treatment of metastatic prostate cancer that is extremely resistant to conventional therapy. Previous work has shown that Palladium complexes can cause cell death by necrosis or apoptosis. This study showed that the Pd complex induced autophagy in some cases, and therefore points to a new area of investigation. Here we present a comprehensive overview of the effects of a novel Palladium complex on cell viability in an extended panel of cell lines and primary cells, including cancer stem cells, and provide first indications of a complex cell death mechanism. Although we have shown that this is a drug with high toxicity, the potential to use it at lower doses in a combination strategy with autophagy modulators, is worth further exploration. Finally, we think that a key message of this study lies in the use of a panel of cell lines alongside patient samples. We have shown that depending on the genetic background of the cells as well as the aggressiveness of the cancer, there are different outcomes to drug treatment. This should be taken into account when testing other palladium drugs and drugs in general, such that one cell line is not overly relied upon, and that patient samples are included in any study of this kind.

## Supporting Information

Figure S1 Graphs of transformed data from Figure 2 following application of the nonlinear regression (curve fit) that represents the log(inhibitor) ‘v’ normalized response, from which the IC50s were calculated (GraphPad Prism software).
(TIF)

Figure S2 Graphs of transformed data from Figure 3 following application of the nonlinear regression (curve fit) that represents the log(inhibitor) ‘v’ normalized response, from which the IC50s were calculated (GraphPad Prism software).
(TIF)

Figure S3 Images of cells treated for flow cytometry cell cycle analysis and for protein lysates.
(TIF)
