# Supplementary figures and images for: Differential Cytotoxic Activity of a Novel Palladium-Based Compound on Prostate Cell Lines, Primary Prostate Epithelial Cells and Prostate Stem Cells
Source: PLoS One. 2013 May 10;8(5):e64278. doi: 10.1371/journal.pone.0064278 (PMC3651166; doi:10.1371/journal.pone.0064278)

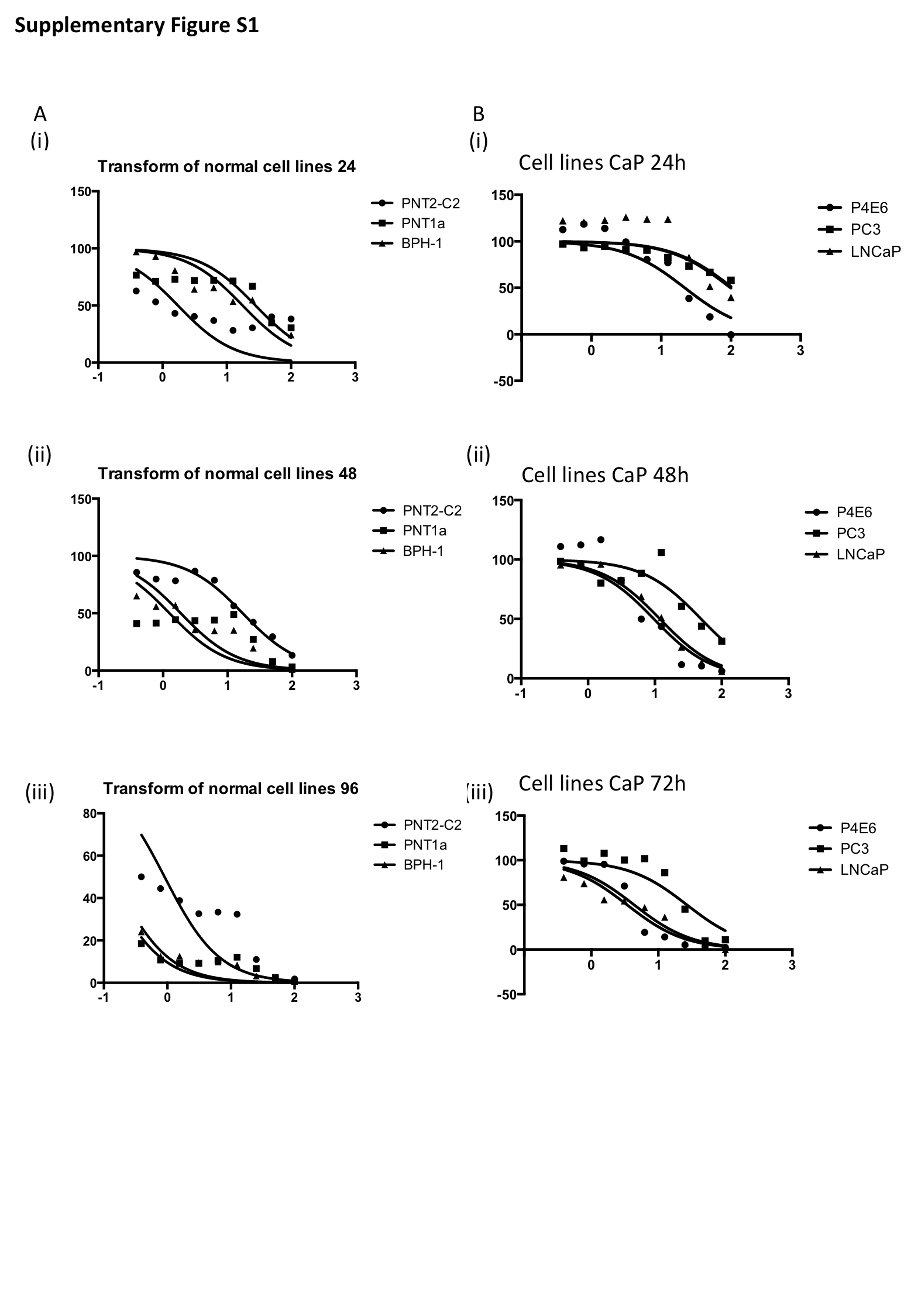

Supplement: Figure S1 — Graphs of transformed data from Figure 2 following application of the nonlinear regression (curve fit) that represents the log(inhibitor) ‘v’ normalized response, from which the IC50s were calculated (GraphPad Prism software). (TIF) [file pone.0064278.s001.tif]

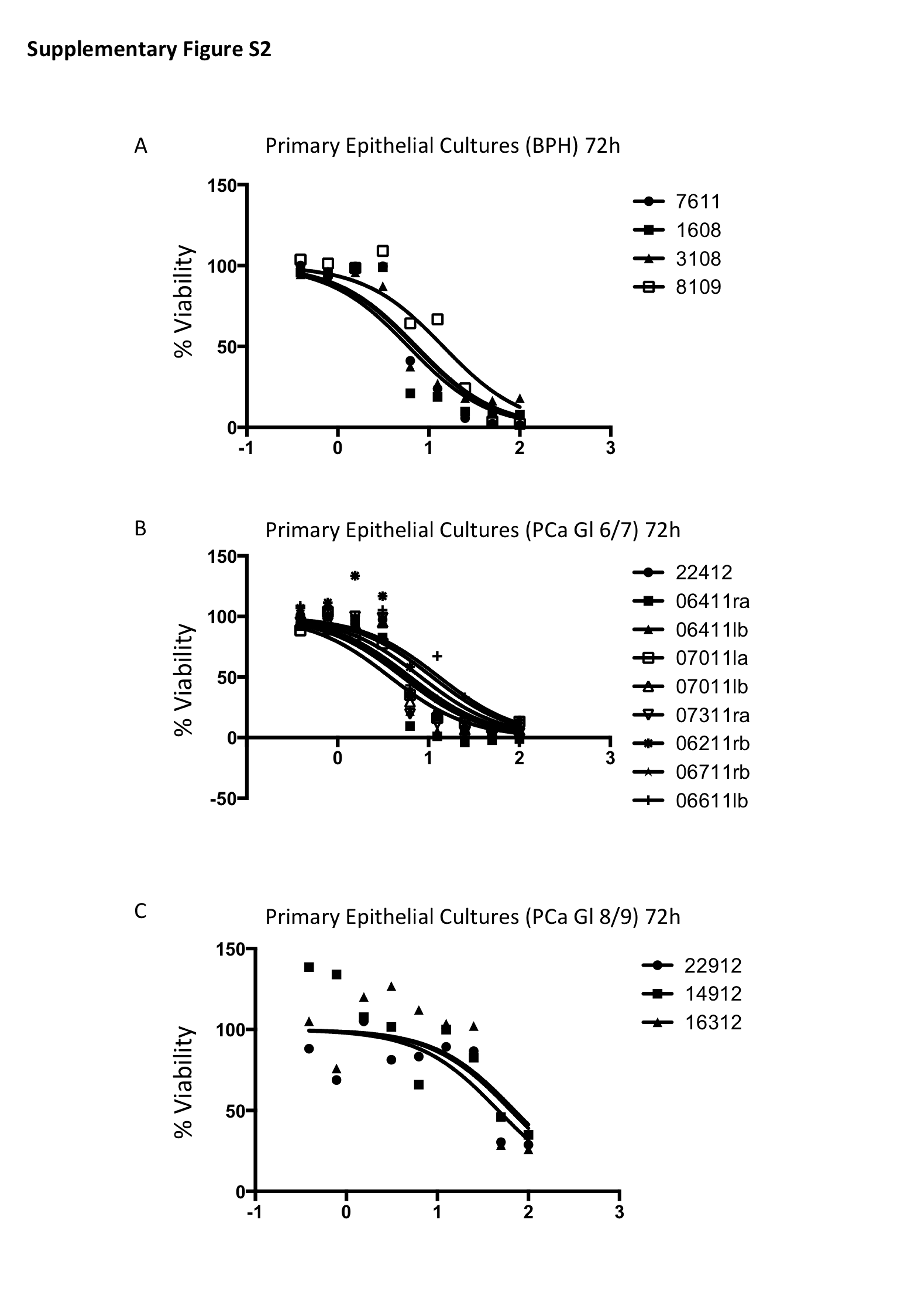

Supplement: Figure S2 — Graphs of transformed data from Figure 3 following application of the nonlinear regression (curve fit) that represents the log(inhibitor) ‘v’ normalized response, from which the IC50s were calculated (GraphPad Prism software). (TIF) [file pone.0064278.s002.tif]

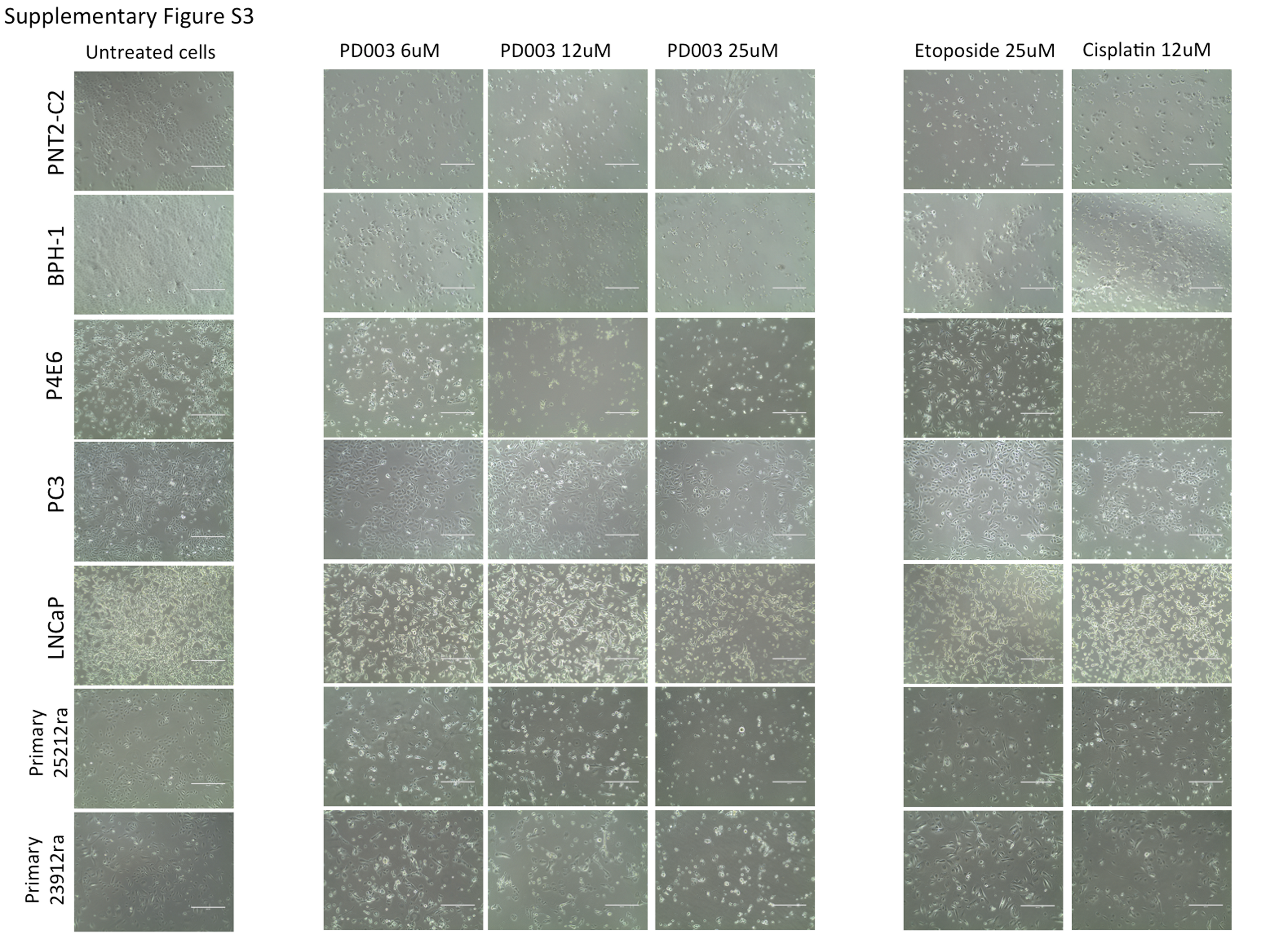

Supplement: Figure S3 — Images of cells treated for flow cytometry cell cycle analysis and for protein lysates. (TIF) [file pone.0064278.s003.tif]
